# Roles of plant hormones and anti-apoptosis genes during drought stress in rice (*Oryza sativa* L.)

**DOI:** 10.1007/s13205-016-0564-x

**Published:** 2016-11-17

**Authors:** Mohammad Ubaidillah, Fika Ayu Safitri, Jun-Hyeon Jo, Sang-Kyu Lee, Adil Hussain, Bong-Gyu Mun, Il Kyung Chung, Byung-Wook Yun, Kyung-Min Kim

**Affiliations:** 1 Division of Plant Biosciences, School of Applied Biosciences, College of Agriculture and Life Science, Kyungpook National University, Daegu, 41566 Korea; 2 Research Institute of Pharmaceutical Sciences, College of Pharmacy, Kyungpook National University, Daegu, 41566 Korea; 3 Department of Agriculture, Abdul Wali Khan University, Mardan, Pakistan; 4 Department of Biotechnology, Catholic University of Daegu, Gyeongsan-Si, Gyeongbuk 38430 Korea

**Keywords:** Anti-apoptosis genes, *AtBI*-1, Drought stress, *OsSAP*, Plant hormone, Rice

## Abstract

We previously identified the rice (*Oryza sativa*) senescence-associated gene *OsSAP* which encodes a highly conserved protein involved in anti-apoptotic activity. This novel Bax suppressor-related gene regulates tolerance to multiple stresses in yeast. Here, we show the effects of drought stress on leaf and root tissues of plants over-expressing *OsSAP* in relation to the levels of phytohormones, abscisic acid (ABA), jasmonic acid (JA), indole-3-carboxylic acid (ICA), gibberellic acid (GA_3_), and zeatin. Results showed that rice plants over-expressing *SAP* were tolerant to drought stress compared to wild type and the plants over-expressing *AtBI*-1, which is a homolog of the human Bax inhibitor-1 in *Arabidopsis*. ABA and JA levels in *OsSAP* and *AtBI*-1 transgenic plants consistently increased up to at least 3 days after drought treatment, whereas lower GA_3_ levels were recorded during early drought period. Comparison between control and transgenic plants overexpressing anti-apoptosis genes *OsSAP* and *AtBI*-1 resulted in different patterns of hormone levels, indicating that these genes are involved in the plant responses to drought stress and present an opportunity for further study on drought stress tolerance in rice and other plant species.

**Electronic supplementary material:**

The online version of this article (doi:10.1007/s13205-016-0564-x) contains supplementary material, which is available to authorized users.

## Introduction

Different abiotic stresses caused by extremes of temperature, salinity, water and nutrient shortage have always been the key limiting factors for agricultural production. These are considered to play a major role in the deterioration of agricultural ecosystems around the world, causing an average 50% reduction in crop yield (Boyer 1982). Most often abiotic stresses mediate early responses during signal transduction and adaptive responses (Kawai-Yamada et al. 2005b). At cellular level, various abiotic stresses lead to a plethora of physiological and biomolecular changes that negatively affect plant productivity (Wang et al. 2001). These changes are mainly the outcome of the overall plant responses aimed at tolerating the stress and prioritizing the most important physiological processes. Among the different abiotic stresses, drought is one of the oldest and longest studied type of stress and perhaps also the most damaging as it affects plants throughout the world, especially in the rainfed areas (Flowers 2004; Zhang et al. 2006; Godfray et al. 2010; Tester and Langridge 2010). The Pro-apoptotic factor BCL2-associated X protein (Bax) plays a central role in regulating PCD (Kroemer 1997) and was first identified as a member of the BCL-2 family of proteins (Oltvai et al. 1993). In mammals, the members of this family are often found as hetero- or homodimers. For example, Bax and BCL2 form a heterodimer which acts as apoptotic activator. Under normal conditions, Bax proteins are located in the cytosol. However, after the initiation of apoptotic signaling Bax proteins undergo conformational changes and are directed to the membranes of different organelles specially the mitochondria (Hsu et al. 1997; Gross et al. 1998; Nechushtan et al. 1999). At the mitochondrial membrane, Bax interacts with and promotes the opening of mitochondrial voltage-dependent anion channel (VDAC) (Shi et al. 2003). This increases the permeability of the mitochondrial outer membranes resulting in caspase activation and the release of cytochrome c and other pro-apoptotic factors (Weng et al. 2005). In plants, the intentional execution of cells in plants also called programmed cell death (PCD) or the hypersensitive response (HR) is a type of defense response that is very important for plant survival, development and adaptation under adverse conditions (Huckelhoven 2004). Interestingly, functional similarity between animal and plant apoptotic machinery also exists. Though Bax genes are yet to be identified in plants, genes that exhibit Bax-like lethality have been found in Arabidopsis (Kawai-Yamada et al. 2001, 2005a). Expression of animal Bax proteins in yeast induced cell death (Sato et al. 1994; Hanada et al. 1995; Lacomme and Cruz 1999; Kawai-Yamada et al. 2001). Similar mechanisms of PCD have also been found in bacteria (Wang and Bayles 2013). This shows that Bax genes and/or Bax-like functionality may be highly conserved across the different kingdoms. Furthermore, homologs of the mammalian Bax inhibitor-1 (BI-1) have also been found in Arabidopsis (Kawai-Yamada et al. 2004), tobacco [*Nicotiana* sp.; (Wang et al. 2001)], and barley [*Hordeum* sp.; (Eichmann et al. 2004)]. Expression of *AtBI*-*1* suppresses mammalian Bax-induced cell death in plants and yeast (Sanchez et al. 2000). *BI*-*1* from pepper conferred resistance to multiple abiotic stresses when expressed in tobacco (Isbat et al. 2009), showing that BI-1 might also play a role in the adaptive responses of plants to abiotic stresses and at the same time keep a check on programmed cell death as both abiotic stresses and PCD are considered detrimental factors in plant development. Both apoptosis in animals and PCD in plants are highly complex processes and the underlying molecular mechanisms are still being investigated. Though Bax-*BI*-*1* interaction presents an intelligent mechanism for regulation/control of PCD in plants, there are many other factors that play important roles in programming cells for intentional suicide. Various aspects of PCD or HR in plants have been elucidated by a significant amount of scientific research available in the literature. Different cellular components/compounds play important roles in PCD such as metal complexes (Arasimowicz-Jelonek et al. 2012; Samuilov et al. 2014; Tewari et al. 2015), reactive oxygen species (ROS) (Foyer 2007a, b; Gadjev et al. 2008; Petrov et al. 2015), nitric oxide (NO) (Arasimowicz-Jelonek et al. 2012), various enzymes such as Cytochrome c (De la Rosa et al. 2015; Gonzalez-Arzola et al. 2015) and caspases (Piszczek and Gutman 2007; Siczek and Mostowska 2012), various lipids and lipid derivatives (Biswas and Mano 2015), secondary metabolites, e.g., chitosan (Vasil’ev et al. 2009), ascorbate (Locato et al. 2009) and many others. Similarly genes that play important roles in regulating PCD in plants encode transcription factors (Yang et al. 2014), antioxidant enzymes (Solovieva et al. 2013), calmodulin-binding proteins, e.g., AtBAG6 (Kang et al. 2006), calcium ion transports, e.g., Arabidopsis PPF1 (Li et al. 2004), housekeeping genes, e.g., actin (Franklin-Tong and Gourlay 2008) and other proteins like the programmed cell death 4 (PDCD4) proteins (Cheng et al. 2013), Fas-associated Factor 1 Ortholog (CaFAF1) proteins (Kim et al. 2009), and several others. Some of the important and/or significant molecular mechanisms involved in regulation of PCD in plants have been comprehensively reviewed by Rantong and Gunawardena (2015), Ma and Berkowitz (2011), Kacprzyk et al. (2011) and Reape et al. (2008). Evidence of programmed cell death during senescence also exists (Zhou et al. 2005; Del Duca et al. 2014). An important but largely un-explored research avenue is the connection between PCD and various plant hormones. Only limited information is available in the literature about the role of plant hormones in PCD for example ethylene due to its role in senescence (Trobacher 2009), and cytokinin (Kunikowska et al. 2013). Plant hormones comprise a very important part of the plant response continuum against biotic and abiotic stresses (Davies 1987, 1995) as well as of the growth and development machinery. Plant hormones play key roles in reprogramming complex physiological processes and stress adaptive strategies (Golldack et al. 2013). Most plant hormones have signature biological functions as well as additive, antagonistic and/or synergistic effects in combination with other hormones and signaling molecules (Aloni et al. 2006), hence making the identification of specific hormone networks much more complex and difficult. We previously identified the rice gene encoding Senescence-Associated Protein (*OsSAP*) exhibiting anti-apoptotic activity under salt stress (Ubaidillah et al. 2015). In this study, we evaluated the PCD-suppressing activity of *OsSAP* and the Arabidopsis Bax Inhibitor homolog *AtBI*-*1* by over-expression of these genes in rice (japonica cv. Ilmi and Ilpum) under drought conditions and in relation to different plant hormones.

## Materials and methods

### Plasmid construction for *OsSAP* and *AtBI*-*1*

*OsSAP* and *AtBI*-*1* were cloned in pBIN19 binary vector between *Sal*I and *Nco*I. Open reading frames were PCR amplified using specific primers containing flanking adapter sequences for *Sal*I and *Nco*I. PCR product and pBIN19 were digested with *Sal*I and *Nco*I restriction enzymes (TaKaRa, Japan) and then ligated using T4 DNA ligase (Promega). All steps were performed according to the manufacturer’s instructions. The pBIN19-*CaMV*35S::*OsSAP*::GFP and pBIN19-*CaMV*35S::*AtBI*-*1*::GFP constructs thus generated were transformed into *Agrobacterium tumefaciens* strain LBA4404 after sequence confirmation (Supplementary Fig. 1).

### *Agrobacterium*-mediated transformation

The pNIB19-*CaMV*35S::*OsSAP*::GFP and pBIN19-*CaMV*35S::*AtBI*-*1*::GFP constructs were transformed into rice through tissue culture technique. Briefly, calli of *Oryza sativa* L. ssp. japonica cv. Ilmi and Ilpum were produced by seed culture in Murashige and Skoog induction medium (4.14 g/L MS vitamin powder, 30 g/L sucrose, 0.3 g/L casein hydrolysate, and 50 mg/L 2,4-D, pH 5.6) at 28 °C under continuous dark for 30 days. *Agrobacteria* containing both the constructs were grown in LB medium containing 20 mg/L spectinomycin, 50 mg/L kanamycin, and 25 mg/L rifampicin at 28 °C until O.D_600_ = 1. The cultured *Agrobacteria* were then pelleted by centrifugation at 3000 rpm for 10 min. The resulting pellet was re-suspended in an equal volume of MS-Acetosyringone (MS-AS) medium (20 g/L sucrose, 10 g/L glucose, 0.3 g/L casein hydrolysate, 100 μM acetosyringone, pH 5.6). These bacteria were then co-incubated with the rice calli for 1 h at 28 °C in a shaking incubator at 180 rpm. After draining out the *Agrobacterium* suspension, the calli were kept on sterile tissue papers and then transferred to MS co-cultivation medium (MS callus induction medium containing 50 mg/L, 2.4-D and 100 µM acetosyringone) and incubated at 28 °C for 3 days in dark. Then, the calli were washed with distilled water twice and 250 mg/L carbenicillin solution. Extra solutions were dried on sterile tissue papers and incubated on MS elimination medium (MS callus induction medium containing 250 mg/L carbenicillin) at 32.5 °C for 10 days in dark. Actively growing calli were transferred to selection and regeneration medium MS basal medium supplemented with 1 mg/L 1-naphthaleneacetic acid, 3 mg/L 6-benzyl adenine, 3 g/L casein hydrolysate, 30 g/L maltose, 250 mg/L carbenicillin, and 40 mg/L geneticin) and incubated under long day light conditions (16 h light and 8 h dark) at 28 °C with continuous sub-culturing on the same medium after 15 days until shoot generation. Regenerated shoots were transferred to rooting medium (half-strength plain MS medium supplemented with 50 mg/L geneticin). Rooted plants were then transferred to soil after 4 weeks. Subsequently, well-established hardened plants were transferred to fresh soil in pots in green house. All transgenic plants were genotyped for the presence of transgene using PCR.

### Polymerase chain reaction (PCR) analysis

Genomic DNA was extracted from leaf tissue of the transgenic plants using DNeasy Plant Mini Kit (QIAGEN, Germany) following manufacturer’s instructions. The primers used for amplifying a 420 bp fragment of *OsSAP* gene were: 5′-CCTTTCCATTTGGGAATCCAGCC-3′ and 5′-GCCCAGGGTTTCACCAGGAAGTT-3′. The PCR conditions used were: 94 °C for 10 min followed by 45 cycles of 94 °C for 30 s, 57 °C for 30 s, 72 °C for 1 min, and a final extension at 72 °C for 5 min. For confirmation of *AtBI*-1 transgene, the primers used were: 5′-ATGGATGCGTTCTCTTCCTT-3′ and 5′-CAGCCCCTCAGTTTCTCCTT-3′, which generated a 720 bp fragment. The PCR conditions used were: 94 °C for 10 min followed by 45 cycles of 94 °C for 30 s, 55.5 °C for 30 s, 72 °C for 1 min, and a final extension at 72 °C for 5 min. The PCR products were visualized after electrophoresis on 1% agarose gels.

### Flanking sequence tags (FSTs) analysis

Genomic DNA from T1 lines was digested with *Bfa*I (NEB, UK) for 5 h at 37 °C. Next 5-10 μl of the restriction digestion reaction was used for ligation of 12.5 pmol *Bfa*I adapter sequences (ADP1; 5′-CTAATACGACTCACTATAGGGCTCGAGCGGCCGGGCAGGT-3′, and ADP2; 5′-TAACCTGCCCAA-3′) to the digested fragments using T4 DNA ligase (NEB, UK) for 11 h at 14 °C. Linear amplification reaction contained whole *Bfa*I digest, 200 nM primers AP1 (5′-GGATCCTAATACGACTCACTATAGGGC-3′), pBIN-TDNA1 (5′-CAATCAGCTGTTGCCCGTCT-3′) for right border, pBIN-TDNA3 (5′-CCAAACGTAAAACGGCTTGT-3′) for left border, dNTPs (250 µM each), and *Taq* DNA polymerase (TaKaRa, Japan). Amplification was performed using the following parameters. Initial denaturation at 94 °C for 2 min, 30 cycles of 94 °C for 35 s, annealing at 64 °C for 1 min, extension at 73 °C for 1.5 min, and final extension at 73 °C for 3 min. Subsequently, 1 µL of the first amplification reaction was used as a template for the second PCR with a reaction mixture containing AP2 (5′-TATAGGGCTCGAGCGGC-3′), nested T-DNA primers pBIN-TDNA2 for right border (5′-GTCTCACTGGTGAAAAGAAA-3′) pBIN-TDNA4 for left border (5′-GGTCATAACGTGACTCCCTTA-3′), and adapter primers. All the products so obtained were sequenced and analyzed through BLASTN search against binary vector sequences and plant genomic sequences.

### Analysis of drought tolerance

Sterilized and chilled wild type and T_1_ transgenic seeds were sown in plastic trays in a greenhouse under long day light conditions at 30 ± 2 °C. Standard agronomic practices were followed for irrigation and fertilizer requirements, etc. When the plants were 5 weeks old, drought stress was imposed by decanting all the water from the trays to speed up dehydration. Further irrigation was stopped for 4 days and sample collection was made on 3 and 4 days after withholding water for plant hormone measurement, because prolonged drought stress more than 5 days under our condition caused completed death of plants. Therefore, data on survival rate were recorded with at least 50 plants of each genotype tested. Leaf and root samples were collected 3 and 4 days post-treatment in liquid nitrogen and stored at −80 °C.

### RNA extraction and cDNA synthesis

Total RNA was isolated from leaf and root tissues using RNeasy Plant Mini Kit (QIAGEN, Germany) following the manufacturer’s protocol. Briefly, 100 mg triplicate samples were taken from control and drought-treated WT, *OsSAP* and *AtBI*-*1*. The samples were crushed in liquid nitrogen and vigorously re-suspended in a microcentrifuge tube using 450 µL buffer RLT on a vortex, followed by incubation at 56 °C for 3 min. The lysate was then transferred to QIA shredder spin column and centrifuged at full speed on a bench-top centrifuge for 2 min. The supernatant of the flow-through was then transferred to a new microcentrifuge tube. Half volume 99% ethanol was added to the tubes and mixed immediately. All the contents were then transferred to RNeasy spin columns and centrifuged for 15 s at 10,000 rpm. After discarding the flow-through, the columns were washed with 700 µL buffer RW1 and then washed with 500 µL buffer RPE. After a final centrifugation for 1 min to remove the remaining liquid, the total RNA was eluted in 30 µL RNase-free water. cDNA was synthesized from 1 µg total RNA using qPCRBIO cDNA synthesis kit according to the manufacturer’s instructions. The reaction mixture [5× synthesis mix (4 µL), reverse transcriptase (1 µL) and nuclease-free water up to 20 µL] was incubated at 27 °C for 10 min and 42 °C for 30 min before final inactivation at 85 °C for 10 min.

### Quantitative measurement of major plant hormones

Plant hormones like abscisic acid (ABA), indole-3 carboxylic acid (ICA), jasmonic acid (JA), gibberellic acid (GA) and zeatin content were measured according to (Ubaidillah et al. 2015). Briefly, a triplicate of fresh plant tissue (200 mg) was prepared in batches for the measurement all plant hormones above and was ground in liquid nitrogen to fine powder with mortar and pestle. The tissue powder was then dissolved in 100 µL of specific working solution of internal standard and 700 µL of extraction solvent (Ubaidillah et al. 2015). The resulting extracts were then through a series of centrifugation and purification step with a mixture of iso-propanol/H_2_O/concentrated HCl (2:1:0.002, v/v/v). The final concentration was re-dissolved in 100 µL methanol and a half was subjected to ESI-triple quadrupole mass spectrometer (HPLC–ESI–MS/MS, Applied Biosystems, USA) equipped with a reverse-phase C18 Gemini column (150 × 2.00 mm, 5-µm particle size, Phenomenex, USA).

### Statistical analysis

All experiments were replicated at least three times, and all data were analyzed using the SPSS program (IBMSPSS Statistics, version22, NC).

## Results

To evaluate the PCD-suppressing activity of *OsSAP* and *AtBI*-*1* in relation to drought stress tolerance in rice and determine their PCD inhibition mechanism, we expressed *OsSAP* and *AtBI*-1 cloned under the control of the *CaMV*35S promoter in rice. In this first generation (*T*_0_), we obtained more than 100 transformants for both the cultivars Ilmi and Ilpum. All these transformants were resistant to kanamycin. All the plants of *T*_0_ generation were genotyped through PCR. After genotyping, 5 independent lines (2 in Ilmi and 3 in Ilpum) containing *CaMV35S::OsSAP* and 20 independent lines (12 in Ilmi and 8 in Ilpum) containing *CaMV35S::AtBI*-*1* were confirmed as proper transgenic lines. No phenotypic differences were observed among the lines expressing either of the transgenes. After the T-DNA flanking region sequencing, *T*_0_ lines expressing a single intergenic copy of either of the transgenes were sown again to harvest *T*_1_ seeds for further studies.

### Response of anti-apoptosis genes to drought stress

**Fig. 1:**
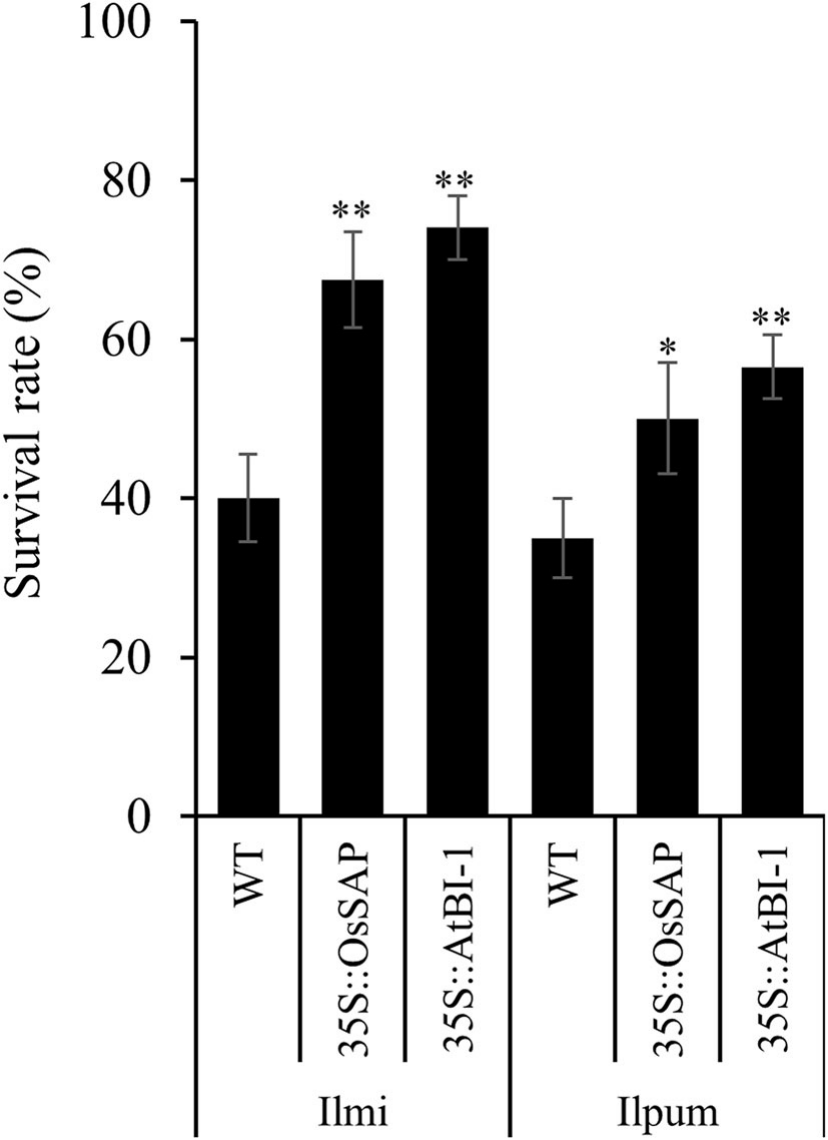
Tolerance of the *OsSAP* and *AtBI*-*1* transformants to drought stress compared with control. *A*, phenotype after drought stress in transgenic rice overexpressing *OsSAP* and *AtBI*-*1*. *L*, Ilmi; *P*, Ilpum; *T*_*1*_ *-LO*, *OsSAP* T_1_ transformants in Ilmi; *T*_*1*_ *-PO, OsSAP* T_1_ transformants n Ilpum; *T*_*1*_ *-LA*, *AtBI*-*1* T_1_ transformants in Ilmi; *T*_*1*_ *-PA*, *AtBI*-*1* T_1_ transformants in Ilpum. *, **Significant at 5 and 1% levels, respectively

T1 transgenic lines over-expressing *OsSAP* and *AtBI*-1 were tested for their response to drought stress. Both types of transgenic lines were resistant to drought stress compared to wild type (WT) plants as shown by significantly higher survival rates. Withering and leaf shrinking were observed in WT plants after 4 days of drought stress. After 5 days, the survival rate of both the transgenic lines in both cultivars was significantly higher than WT plants (Fig. 1). Survival rates were measured after 5 days and are represented by mean values for at least 50 transformants. Furthermore, *AtBI*-*1* over-expression increased the survival rates of plants from both the cultivars compared to *OsSAP* and WT plants. However, in general the survival rates of the cultivar Ilmi were approx. 20% higher than those of Ilmi indicating a higher endogenous tolerance to drought stress compared to the Ilpum cultivar. The survival rate of *OsSAP*-Ilmi transformants was 10% higher than that of WT-Ilmi plants. Similarly, the survival rate of *OsSAP*-Ilpum transformants was 15% higher than that of WT-Ilpum (Fig. 1).

**Fig. 2:**
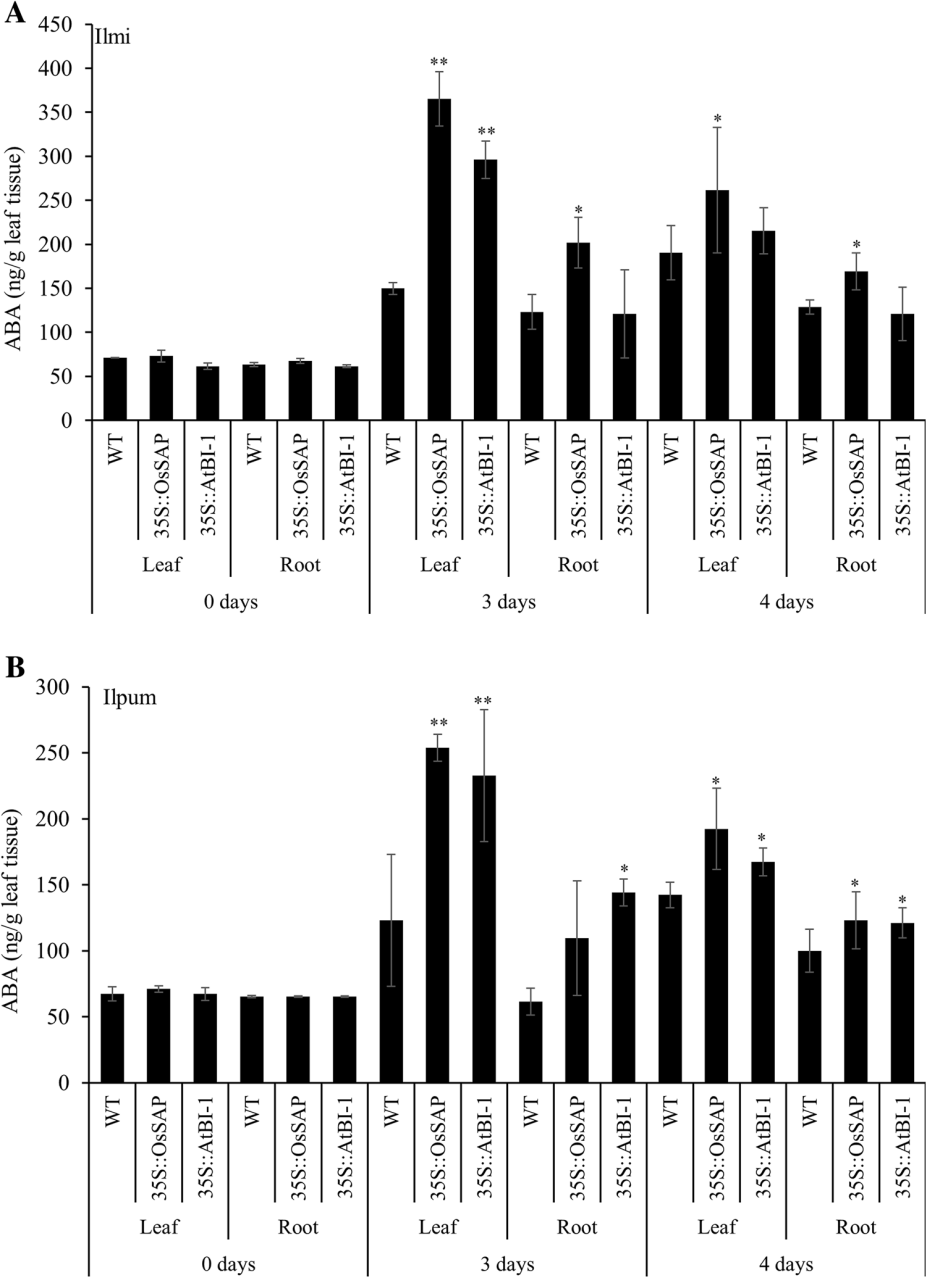
Abscisic acid (ABA) level in transformants compared with non-transformant controls under drought conditions overtime. Mean values ± SE are given. LL: control Ilmi-leaf, *LR*, control Ilmi-leaf; *PL*, control Ilpum-leaf; *PR*, control Ilpum-root; *LOL*, *OsSAP* Ilmi transformants-leaf; *LOR*, *OsSAP* Ilmi transformants-root; *POL*, *OsSAP* Ilpum transformants-leaf; *POR*, *OsSAP* Ilpum transformants-root; *LAL*, *AtBI*-*1* Ilmi transformants-leaf; *LAR*, *AtBI*-*1* Ilmi transformants-root; *PAL*, *AtBI*-*1* Ilpum transformants-leaf; *PAR*, *AtBI*-*1* Ilpum transformants-root. *, **Significant at 5 and 1% levels, respectively

The transgenic lines expressing either of the transgenes were resistant to drought stress. This prompted us to measure the contents of various plant hormones, such as abscisic acid (ABA), jasmonic acid (JA), gibberellic acid (GA_3_), zeatin, and indole 3 carboxylic acid (ICA) in the leaf and root tissues before and after drought stress treatment. Results showed that *OsSAP* and *AtBI*-*1* over-expression significantly increased the ABA contents in the leaves and roots of both the cultivars under study for up to 4 days after drought stress induction (Fig. 2).

**Fig. 3:**
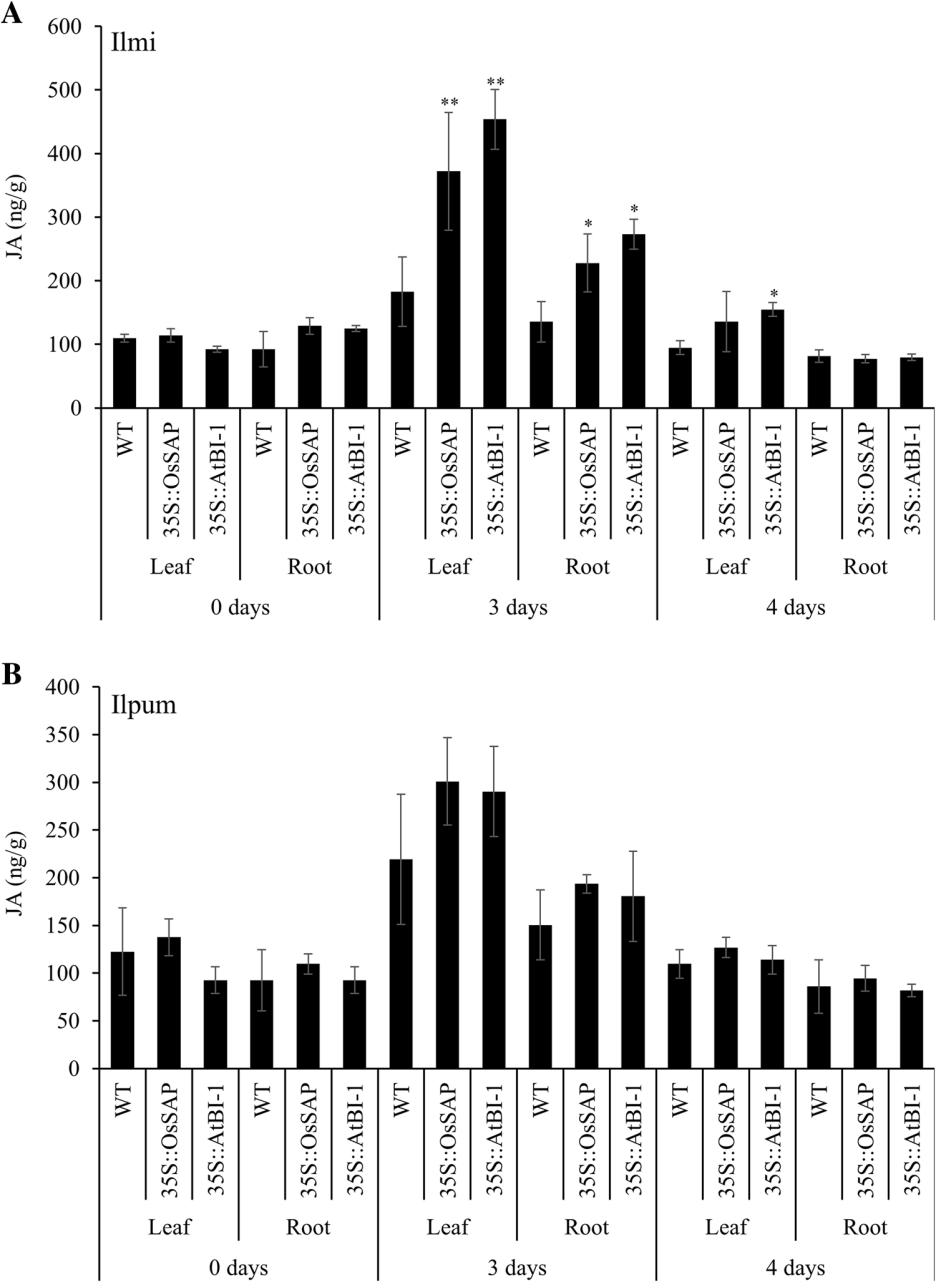
Jasmonic acid (JA) level in transformants compared with non-transformant controls under different drought conditions. Mean values ± SE are given. *LL*, control Ilmi-leaf; *LR*, control Ilmi-leaf; *PL*, control Ilpum-leaf; *PR*, control Ilpum-root; *LOL*, *OsSAP* Ilmi transformants-leaf; *LOR*, *OsSAP* Ilmi transformants-root; *POL*, *OsSAP* Ilpum transformants-leaf; *POR*, *OsSAP* Ilpum transformants-root; *LAL*, *AtBI*-*1* Ilmi transformants-leaf; *LAR*, *AtBI*-*1* Ilmi transformants-root; *PAL*, *AtBI*-*1* Ilpum transformants-leaf; *PAR*, *AtBI*-*1* Ilpum transformants-root. *, **Significant at 5 and 1% levels, respectively

*OsSAP* and *AtBI*-*1* overexpression significantly increased JA levels in both rice cultivars, especially in the leaves after 3 days of drought stress induction. However, JA levels dropped dramatically on after 4 days of drought stress induction (Fig. 3). JA levels in control Ilmi and Ilpum plants increased after 3 days in both leaf and root tissues, but decreased after 4 days of drought stress treatment. Different patterns were observed in Ilmi transgenic lines overexpressing *OsSAP* and *AtBI*-1. The JA level in leaf tissue after 3 days of treatment increased dramatically by approximately fivefold compared to the JA level before treatment. In root tissue, the level increased approximately threefold after drought stress treatment, whereas in control rice only a slight increase of JA levels was noted. The level of JA in Ilpum transgenic lines overexpressing *OsSAP* and *AtBI*-1 also increased, although to a lesser degree. The level of JA in Ilpum transgenic lines overexpressing *OsSAP* and *AtBI*-1 increased by 3- and 2-fold in leaf and root tissue, respectively, after 3 days of drought stress. JA level decreased after 4 days of treatment in all genotypes and there was no difference between transgenic lines and control rice plants (Fig. 3).

**Fig. 4:**
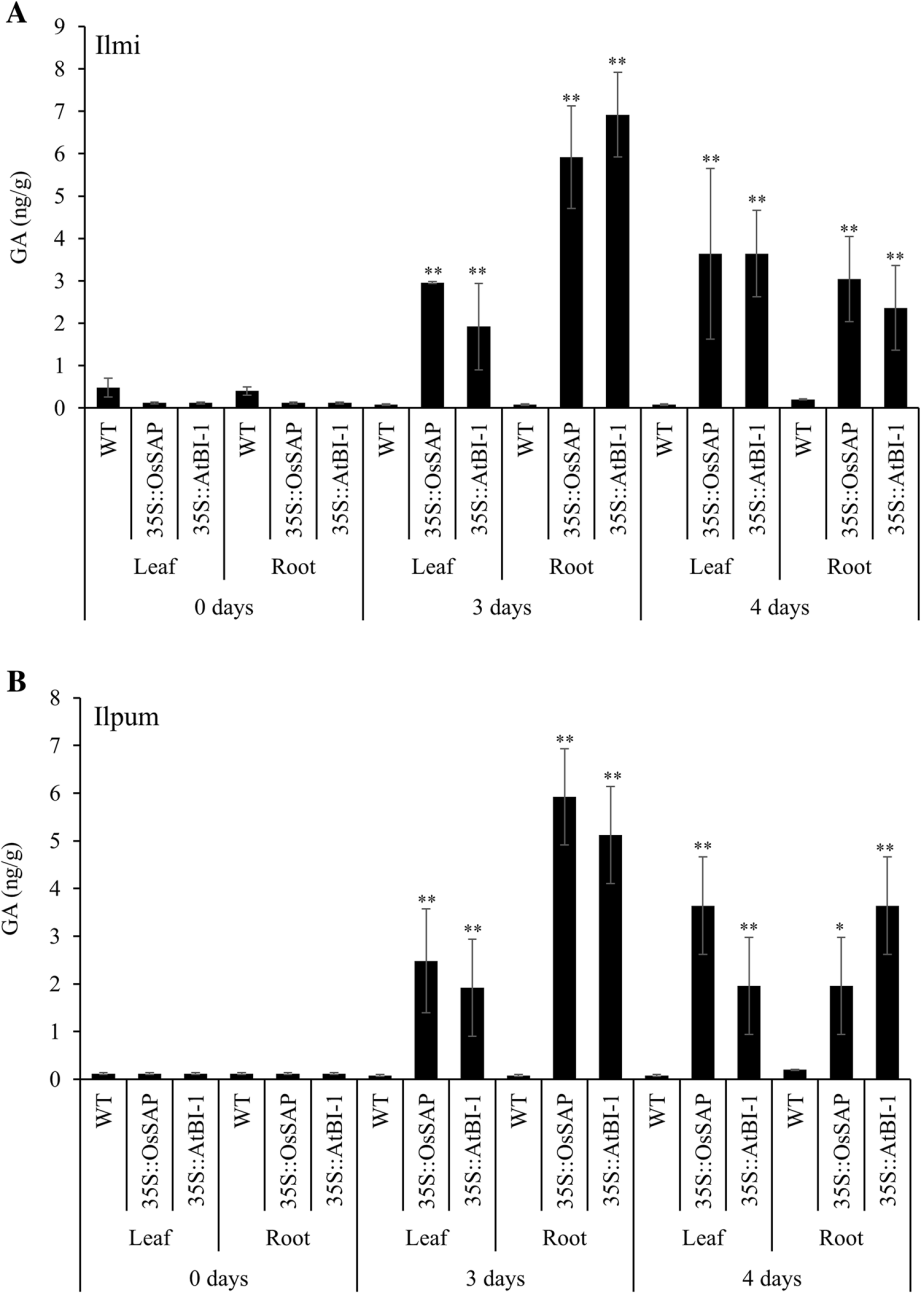
Gibberellic acid (GA_3_) level in transformants compared with non-transformant controls under different drought conditions. Mean values ± SE are given. *LL*, control Ilmi-leaf; *LR*, control Ilmi-leaf; *PL*, control Ilpum-leaf; *PR*, control Ilpum-root; *LOL*, *OsSAP* Ilmi transformants-leaf; *LOR*, *OsSAP* Ilmi transformants-root; *POL*, *OsSAP* Ilpum transformants-leaf; *POR*, *OsSAP* Ilpum transformants-root; *LAL*, *AtBI*-*1* Ilmi transformants-leaf; *LAR*, *AtBI*-*1* Ilmi transformants-root; *PAL*, *AtBI*-*1* Ilpum transformants-leaf; *PAR*, *AtBI*-*1* Ilpum transformants-root. *, **Significant at 5 and 1% levels, respectively

GA_3_ levels were significantly lower in all the wild type and transgenic lines under normal conditions. However, these levels significantly increased after 3 and 4 days of drought stress induction in both the leaves and roots of Ilmi and Ilpum plants (Fig. 4). GA_3_ levels in leaf and root tissues of the transgenic plants were 2–7 fold higher than those of the control plants, although this level was not maintained throughout the prolonged drought stress conditions. GA_3_ levels in Ilmi and Ilpum transgenic lines overexpressing *AtBI*-1 were similar to those in *OsSAP* transgenic lines (Fig. 4).

**Fig. 5:**
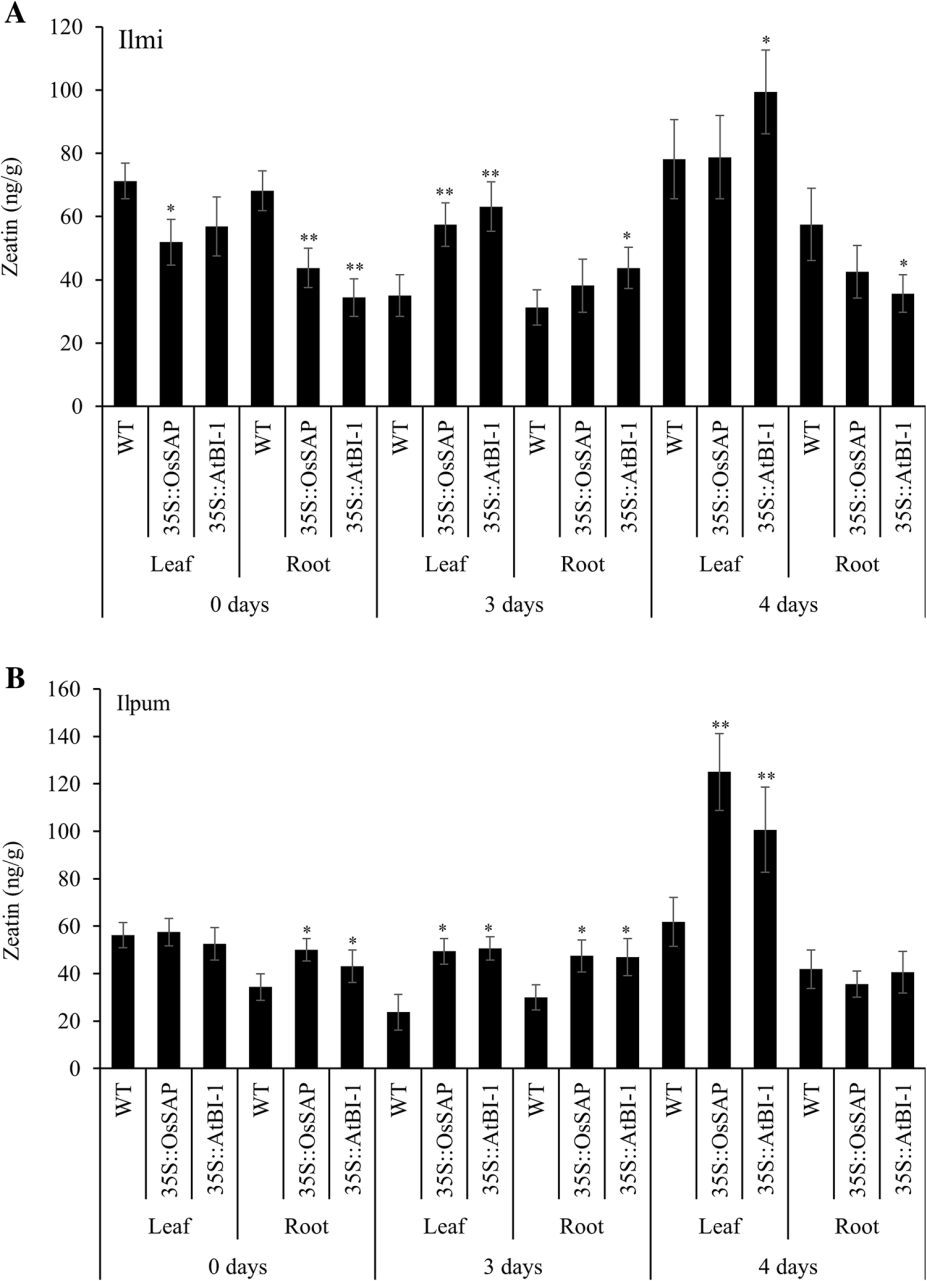
Zeatin level in transformants compared with controls under different drought conditions. Mean values ± SE are given. *LL*, control Ilmi-leaf; *LR*, control Ilmi-leaf; *PL*, control Ilpum-leaf; *PR*, control Ilpum-root; *LOL*, *OsSAP* Ilmi transformants-leaf; *LOR*, *OsSAP* Ilmi transformants-root; *POL*, *OsSAP* Ilpum transformants-leaf; *POR*, *OsSAP* Ilpum transformants-root; *LAL*, *AtBI*-*1* Ilmi transformants-leaf; *LAR*, *AtBI*-*1* Ilmi transformants-root; *PAL*, *AtBI*-*1* Ilpum transformants-leaf; *PAR*, *AtBI*-*1* Ilpum transformants-root. *, **Significant at 5 and 1% levels, respectively

Estimation of the cytokinin zeatin levels under drought conditions revealed increased levels of zeatin in both the control and transgenic lines. A significant increase in zeatin levels was observed in the leaf and root tissues after 3 and 4 days of drought stress induction in all the transgenic lines of ilmi and ilpum (Fig. 5). Prolonged drought stress increased zeatin levels in all genotypes.

**Fig. 6:**
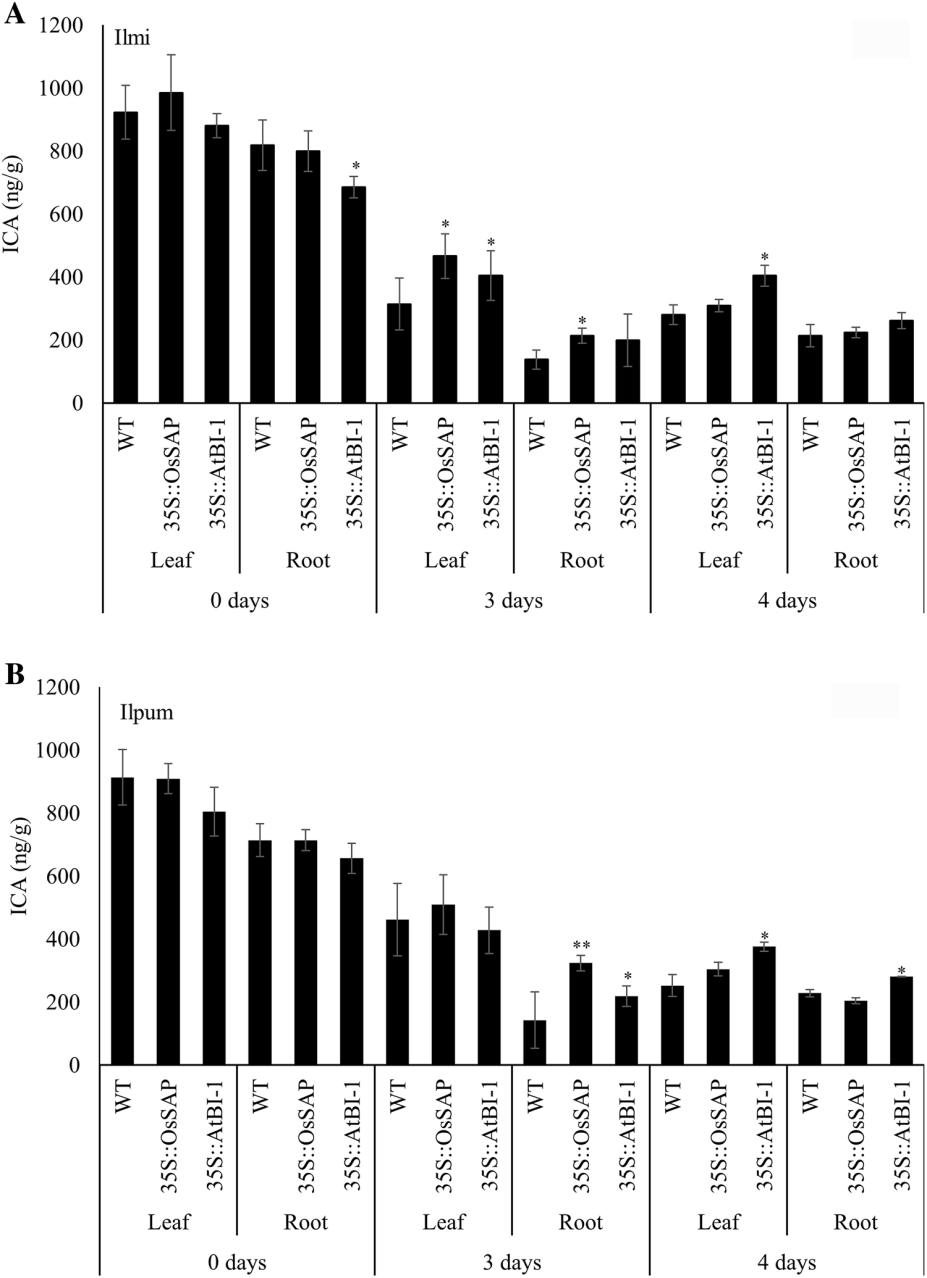
Indole-3-carboxylic acid (ICA) level in transformants compared with non-transformant controls under different drought conditions. Mean values ± SE are given. *LL*, control Ilmi-leaf; *LR*, control Ilmi-leaf; *PL*, control Ilpum-leaf; *PR*, control Ilpum-root; *LOL*, *OsSAP* Ilmi transformants-leaf; *LOR*, *OsSAP* Ilmi transformants-root; *POL*, *OsSAP* Ilpum transformants-leaf; *POR*, *OsSAP* Ilpum transformants-root; *LAL*, *AtBI*-*1* Ilmi transformants-leaf; *LAR*, *AtBI*-*1* Ilmi transformants-root; *PAL*, *AtBI*-*1* Ilpum transformants-leaf; *PAR*, *AtBI*-*1* Ilpum transformants-root. *, **Significant at 5 and 1% levels, respectively

The levels of auxin ICA in WT and transgenic lines were measured after drought stress induction. The level of ICA in control rice and transformants did not show a notable difference. Auxin ICA levels in control plants dramatically decreased in leaf and root tissue of all the genotypes after 3 days of drought stress and continued up to 4 days (Fig. 6).

## Discussion

Previously we reported that the over-expression of the anti-apoptotic genes *OsSAP and AtBI*-*1* in rice promoted salt tolerance (Ubaidillah et al. 2015). In this study, we investigated the role of these anti-apoptotic genes in drought stress tolerance and biosynthesis and signaling of various plant hormones which are involved in the regulation of multiple biotic and abiotic stresses. We constitutively over-expressed both the genes under the control of *CaMV35S* promoter. Overall, the over-expression of both the genes promoted drought tolerance of rice cultivars Ilmi and Ilpum. Survival rates of plants after drought stress treatment were also higher for the transgenic lines than WT plants. Involvement of *BI*-*1* related genes inducible by various abiotic and redox stress conditions has been reported in the literature (Watanabe and Lam 2008).

*OsSAP* which encodes senescence-associated protein might be related to the Bax inhibitor gene because the plant *BI*-1 genes are also expressed in diverse tissue types and their expression levels are enhanced during aging and senescence (Eichmann et al. 2004). This study shows that the over-expression of *OsSAP* and *AtBI*-*1* plays a key role in drought stress regulation in rice.

In this study, the alteration of hormone level in transformants overexpressing the anti-apoptosis genes *AtBI*-1 and *OsSAP* was also documented. Transformants harboring *AtBI*-1 and *OsSAP* were used in the analysis. The alteration of hormone levels in rice represents the distinct plant responses toward abiotic stress due to different defence mechanisms. Phytohormones are in a prominent position, and play important regulatory roles in plant physiology that affects developmental processes during abiotic stresses (Cheng et al. 2013; Browse 2009). Three hormones (ABA, JA, and GA_3_) related with defence mechanisms during abiotic stress, particularly drought, were investigated. The role of ABA, JA, and SA as primary signals in the regulation of plant defence has been well established (Bari and Jones 2009; Pieterse et al. 2009). These hormones generate a signal transduction network that leads to a cascade of events responsible for the physiological adaptation of plants to stress. It was evident that the ABA level under drought stress increased in all leaf and root tissues in both *OsSAP* and *AtBI*-1 transformants. It is known that ABA positively contributes toward adaptation to osmotic stress, a major component of several abiotic stresses (Kissoudis et al. 2014). Its role in defence-related responses has been well discussed in the reviews (Ton et al. 2009). ABA in plant cells is maintained dynamically during the processes of synthesis, degradation and transport, and hence by maintaining the endogenous ABA levels, plants maintain their developmental stages along with their response to stress conditions (Kissoudis et al. 2014). Stimulation of the anti-apoptotic proteins’ production is also attributed to ABA which minimizes the expression of these proteins (Scarfì et al. 2009). ABA plays an antagonistic role in response to Reactive Oxygen Species (ROS), including cell death, whereas ABA-treated cells maintain their ability to scavenge ROS (Wang et al. 2008). A similar protective role of ABA against cell death has been observed during androgenesis in developing barley anthers (Wang et al. 2008). It is now understandable that anti-apoptosis and ABA with *AtBI*-1 and *OsSAP* overexpression could have connected pathways.

In the investigation of JA levels in the transformants overexpressing *OsSAP and* *AtBI*-1, and a control, high accumulations of JA under drought stress occurred in *OsSAP* and *AtBI*-1 transformants after moderate stress, but dramatically decreased after prolonged periods of stress. Jasmonic acid (JA) is a well-known signaling molecule in plant defence and stress responses (Hoeberichts and Woltering 2003). The participation of JA in response to abiotic stress, such as drought, has been reported in several species. Stimulated water stress increased endogenous content of JA, followed by the synthesis of jasmonate-induced proteins (Lehmann et al. 1995). It has been reported that tomato cultivars differ in salt tolerance due to different basal JA content. Steady-state amounts of JA and related compounds were higher with drought stress tolerance (Pedranzani et al. 2003). Furthermore, it was suggested that JA-related responses are directly associated with a reset, downstream of gene expression in the biosynthesis pathway (Thines et al. 2007). Together, these studies indicate that JA is an important component of a pathway that negatively regulates cell death and lesion formation. JA is believed to cause this effect by attenuating the O3-induced ROS production, as wounding or treatment of plants with JA has been shown to reduce O3-induced cell death and O3-induced ROS levels (Overmyer et al. 2000). However, the precise mechanisms by which JA signaling regulates anti-apoptosis remain to be elucidated. The response of altered JA levels in the *OsSAP* transformants during stress suggests the involvement of *OsSAP* in the JA physiological pathway. Under drought conditions, plants have developed complex mechanisms to perceive external signals, allowing them to optimally respond to the environment.

Some plant hormones, such as ABA, SA, and JA, regulate protective responses of plants against both abiotic and biotic stresses via synergistic and antagonistic actions, which are referred to as signaling crosstalk (Fujita et al. 2006). In *Arabidopsis*, it has been proposed that both JA and ABA act in the response to moderate drought (30% field capacity; Aimar et al. 2011). JA and ABA might be connected in different stages of the response, driving an acclimation process during growth through an extensive genetic reprogramming to finally reach a new homeostasis (Harb et al. 2010). Overexpression of *OsSAP* and *AtBI*-1 can improve the endogenous level of ABA and JA during the early stages of moderate drought. We suggest that these anti-apoptosis genes involved in increasing the endogenous ABA and JA levels could sufficiently stimulate the preparatory response needed for drought acclimation.

Investigation of GA_3_ level in transformed rice overexpressing *OsSAP* and *AtBI*-1 under drought stress revealed high accumulations of this hormone; however, GA_3_ level was not detected or was at a very low level in the control. These results suggested that GA_3_ is involved only in the response to drought stress. Moreover, altered levels of GA_3_ due to the overexpression of anti-apoptosis genes *OsSAP* and *AtBI*-1 confirmed their roles in the drought stress tolerance mechanism. In particular, convergence and functional modulation of ABA signaling by GA_3_ have a key regulatory function in the plant cellular network of stress and developmental signaling (Golldack et al. 2013). Recently, interesting evidence has been provided for a convergence and crosstalk of GA_3_ and ABA signaling with the developmental regulator JA in plant responses to drought (Wasternack and Hause 2013). It was also reported that exogenous application of GA_3_ can reduce cell damage and improve growth of maize (*Zea mays*) seedlings subjected to water stress (Wang et al. 2008). Therefore, we suggest that overexpression of anti-apoptosis genes *OsSAP* and *AtBI*-1 increase the levels of endogenous GA_3_. GA_3_ was demonstrated to be effective in alleviating cell damage of rice subjected to drought stress. Thus, the results imply that *OsSAP* and *AtBI*-1 can increase GA_3_ level and might help to maintain cell membrane stability and increase tolerance to drought stress. Observation of cytokinin zeatin levels in transformed rice overexpressing *OsSAP* and *AtBI*-1 under moderate drought stress revealed decreased accumulation levels of this hormone in all genotypes, and a slight increase after a prolonged period of stress. We found that zeatin levels were higher in transformant plants compared to those of control plants under drought stress. Decreased cytokinin content in response to drought stress has been observed and leaf zeatin concentration declined under osmotic stress in the tomato (*Solanum lycopersicum*; Walker and Dumbroff 1981). This also indicated that the relationship between zeatin and *OsSAP* and *AtBI*-1 overexpression with anti-apoptosis could have connected pathways. ICA accumulation was different in leaf and root tissues. Drought stress dramatically decreased ICA levels, but no difference between control and transformants plants was observed. However, auxin is involved in the attenuation of defense responses in plants. Conversely, blocking auxin responses increases resistance in plants (Bari and Jones 2009). In summary, this finding suggests that these hormones and response to abiotic stress in rice are not related to each other. Although similar pattern is observed for many components of the pathway along with a number of genes induced by the abiotic stress, it is worth to be noted that the expression pattern of a specific gene varies according to the time and level of its expression under specific experimental conditions in different tissues. The variation that occurs can be attributed to the variation in treatments, their severity and duration. The crosstalk and regulatory mechanisms of different pathways in the plants can also be affected by their hormonal status under variable stress conditions. However, the plant hormones’ regulatory network and its signaling under abiotic stress are yet to be unveiled. Also the stress-related genes involved in hormone signaling are yet to be discovered. Rice transformants overexpressing the anti-apoptosis genes under drought stress having changes in endogenous hormone levels would allow better understanding of the response of plants to drought stress and help develop drought-tolerant crops.

### Proposed model on crosstalk of hormonal signaling in plants

**Fig. 7:**
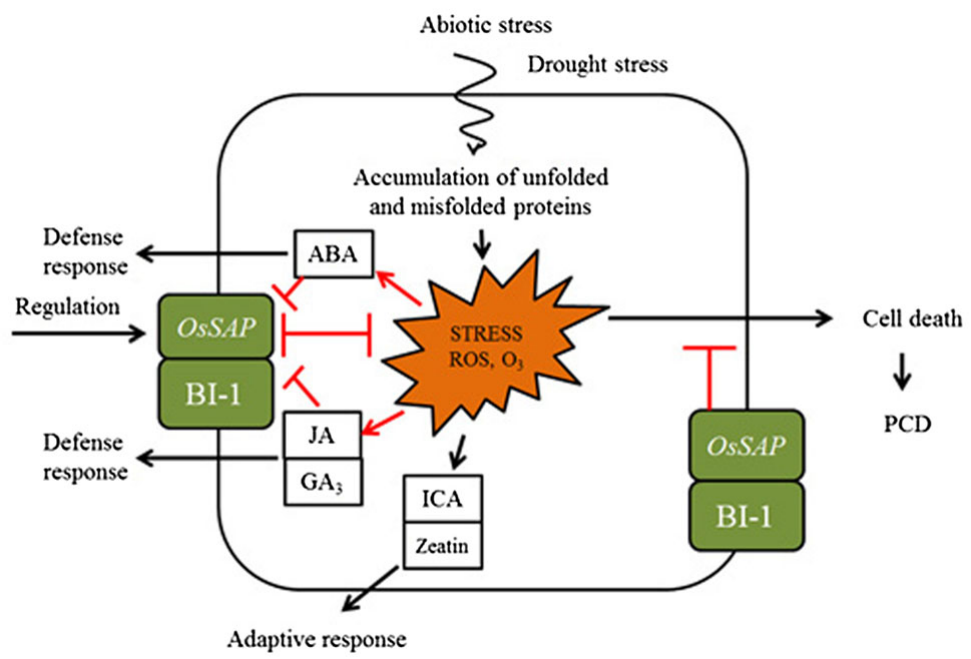
Proposed model on crosstalk of abscisic acid (ABA), gibberellic acid (GA_3_), and jasmonate (JA) signaling in cellular response of plants to drought, elicited by *OsSAP* and *AtBI*-1 genes

In this study, anti-apoptosis genes *OsSAP* and *AtBI*-1 were demonstrated to induce different patterns of hormone levels, indicating that these genes and plant hormone pathways are mechanistically connected in the response of plants to abiotic stress, in particular drought stress. Our hypothesis linking *OsSAP* and *AtBI*-1 genes with hormone signaling in rice plants is schematically shown in Fig. 7.

## Conclusion

*OsSAP*, an anti-apoptotic activator, modulates a diverse plant hormonal signaling network primarily including antagonistic mode of ABA, JA and GA_3_. Particularly, GA_3_ exhibited retarded accumulation kinetics during early stage of drought stress compared to other hormones. Differential accumulation and kinetics mode of plant hormones mediated by the overexpression of *OsSAP* and *AtBI*-*1*, a homolog of the human Bax inhibitor-1 in *Arabidopsis*, would pave a new way to understanding the defense signaling in response to diverse stress condition.

## Electronic supplementary material

Below is the link to the electronic supplementary material.

**Supplementary Fig.** **1** *OsSAP* and *AtBI*-*1* were cloned in pBIN19 binary vector between *Sal*I and *Nco*I. Open reading frames were PCR amplified using specific primers containing flanking adapter sequences for *Sal*I and *Nco*I
