# Supplementary figures and images for: Roles of plant hormones and anti-apoptosis genes during drought stress in rice (Oryza sativa L.)
Source: 3 Biotech. 2016 Nov 17;6(2):247. doi: 10.1007/s13205-016-0564-x (PMC5114211; doi:10.1007/s13205-016-0564-x)

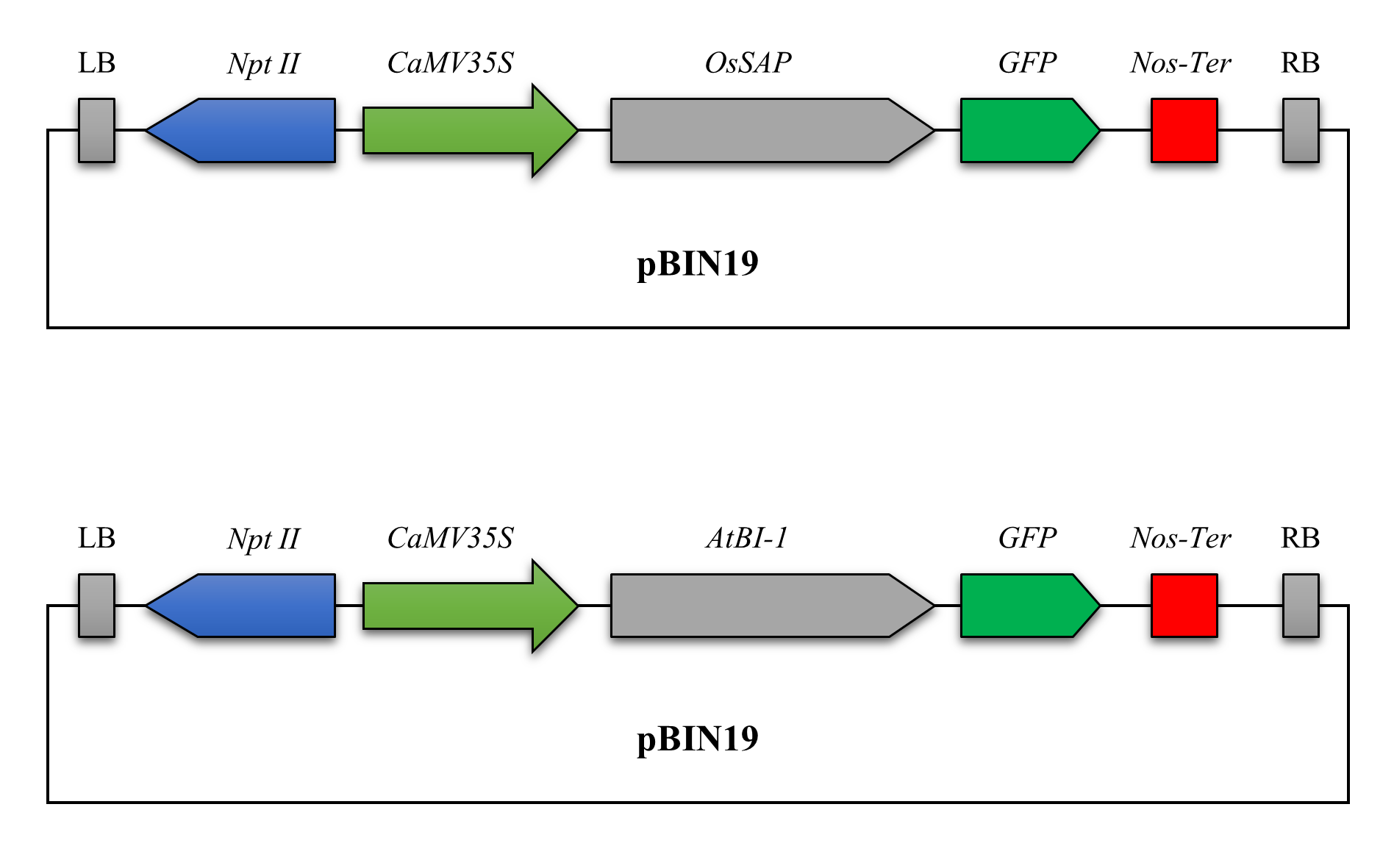

Supplement: Supplementary file 1 — Supplementary Fig. 1 OsSAP and AtBI-1 were cloned in pBIN19 binary vector between SalI and NcoI. Open reading frames were PCR amplified using specific primers containing flanking adapter sequences for SalI and NcoI [file 13205_2016_564_MOESM1_ESM.tif]
